# Combinatorial efficacy of Manuka honey and antibiotics in the *in vitro* control of staphylococci and their small colony variants

**DOI:** 10.3389/fcimb.2023.1219984

**Published:** 2023-10-19

**Authors:** Jiawei Liang, Mowalolaoluwa Adeleye, Laura A. Onyango

**Affiliations:** Department of Biology, Faculty of Natural and Applied Sciences, Trinity Western University, Langley Township, BC, Canada

**Keywords:** *Staphylococci*, small colony variants, manuka honey, combinatorial treatment, antibiotic resistance, non-antibiotic alternatives

## Abstract

**Introduction:**

Staphylococci are among the list of problematic bacteria contributing to the global antibiotic resistance (ABR) crisis. Their ability to adopt the small colony variant (SCV) phenotype, induced by prolonged antibiotic chemotherapy, complicates staphylococcal infection control options. Novel and alternative approaches are needed to tackle staphylococcal infections and curb ABR. Manuka honey (MH), a non-antibiotic alternative is recognized for its unique antibacterial activity based on its methylglyoxal (MGO) component.

**Methods:**

In this study, MH (MGO 830+) was tested in combination with gentamicin (GEN), rifampicin (RIF), or vancomycin (VA) against staphylococcal wildtype (WT) and SCVs. To our knowledge, there are no current studies in the literature documenting the effects of MH on staphylococcal SCVs. While *Staphylococcus aureus* is well-studied for its international ABR burden, limited data exists demonstrating the effects of MH on *S. epidermidis* and *S. lugdunensis* whose pathogenic relevance and contribution to ABR is also rising.

**Results & discussion:**

The three staphylococci were most susceptible to RIF (0.06-0.24 μg/ml), then GEN (0.12-0.49 μg/ml), and lastly VA (0.49-0.96 μg/ml). The MICs of MH were 7%, 7-8%, and 6-7% (w/v), respectively. Fractional inhibitory concentration (FIC) evaluations showed that the combined MH + antibiotic effect was either additive (FICI 1-2), or partially synergistic (FICI >0.5-1). While all three antibiotics induced SCVs *in vitro*, stable SCVs were observed in GEN treatments only. The addition of MH to these GEN-SCV-induction analyses resulted in complete suppression of SCVs (p<0.001) in all three staphylococci, suggesting that MH’s antibacterial properties interfered with GEN’s SCV induction mechanisms. Moreover, the addition of MH to growth cultures of recovered stable SCVs resulted in the inhibition of SCV growth by at least 99%, indicating MH’s ability to prevent subsequent SCV growth. These *in vitro* analyses demonstrated MH’s broad-spectrum capabilities not only in improving WT staphylococci susceptibility to the three antibiotics, but also mitigated the development and subsequent growth of their SCV phenotypes. MH in combination with antibiotics has the potential to not only resensitize staphylococci to antibiotics and consequently require less antibiotic usage, but in instances where prolonged chemotherapy is employed, the development and growth of SCVs would be hampered, providing a better clinical outcome, all of which mitigate ABR.

## 1 Introduction

The discovery of penicillin and subsequent antibiotics has revolutionized the fields of human and veterinary medicine, agriculture, and aquaculture over the past seven decades. Nonetheless, the abuse and overuse of antibiotics have accelerated the global rise of antibiotic-resistant bacteria (ARB) and rendered the infections they cause difficult to treat (Meek et al., 2015). Antibiotic resistance (ABR) has been classified as a dire public health issue that threatens to return society to a pre-antibiotic era, projected to cost the world’s economy US$100 trillion by the year 2050 (WHO, 2014; Courvalin, 2016; Roope et al., 2019). Although staphylococci are commensals of the skin and mucus membranes of healthy individuals, their opportunistic nature is initiated when host defenses are compromised, resulting in mild to life-threatening infections, some of which present with significant antibiotic resistance patterns, contributing to their involvement among the list of problematic ARB (Crossley et al., 2009). *Staphylococcus aureus*, a coagulase-positive staphylococci (CPS) is well known for its ability to invade wounds and surgical sites, can lead to bacterial sepsis, prosthetic valve endocarditis, and post-neurosurgical meningitis, to mention a few, and is associated with increasingly high treatment costs and mortality rates (Lowy, 1998; Crossley et al., 2009; Liu et al., 2015). Methicillin-resistant *Staphylococcus aureus* (MRSA) is globally renowned for its role in both community-associated and health-care-associated infections. In nosocomial settings, it is especially problematic to resolve, leading to extended hospitalizations, elevated medical costs, and increased mortality rates (Livermore, 2000; Klevens et al., 2006). *Staphylococcus epidermidis*, *Staphylococcus lugdunensis*, and *Staphylococcus haemolyticus* are examples of problematic coagulase-negative staphylococci (CNS) that have gained clinical recognition for their association with infections of indwelling medical devices, examples of which include pacemaker infections, prosthetic valve endocarditis, and infections involving vascular grafts (Kloos and Bannerman, 1994; John and Harvin, 2007; Dupont et al., 2010; Carpaij et al., 2011).

ABR is further complicated by the ability of bacteria, like the staphylococci, to form small colony variant (SCV) phenotypes, a unique bacterial sub-population that, in comparison to wildtype (WT) strains, display a range of atypical characteristics that not only hamper their clinical identification but complicate their pathogenesis (Onyango et al., 2008; Onyango and Alreshidi, 2018). Their reduced metabolic activity, down-regulated virulence mechanisms, heightened biofilm formation, and extensive tolerance to many clinically important antibiotics often impede their detection and subsequent clearance efforts of circulating host immune components and administered antibiotics (Häußler, 2004; Tuchscherr et al., 2016). SCVs also employ a phenotypic switching mechanism (PSM) transitioning between highly resilient phenotypes when challenges persist and generating virulent WT phenotypes when pressures abate. This PSM maintains reservoirs, enhances recalcitrance, and undermines treatment options using conventional methods where staphylococcal SCV infections are concerned (Tuchscherr et al., 2011; Onyango and Alreshidi, 2018).

Overall, there’s an urgent need for more effective antimicrobial alternatives to complement current chemotherapeutics in tackling problematic infections that perpetuate the ABR crisis. Due to the rapid development of resistance mechanisms, an important research objective in this pursuit is to explore non-antibiotic alternatives. Honey is one such alternative with a longstanding therapeutic history in ancient and traditional medicine, owing to its multifactorial antimicrobial properties. In recent times, honey has primarily been employed in topical applications to treat a range of wound infections, many of which were recalcitrant to conventional antibiotic therapy, and sometimes involved multidrug- resistant organisms (Maddocks and Jenkins, 2013; Israili, 2014; Cooke et al., 2015; Carter et al., 2016; Combarros-Fuertes et al., 2020; Nolan et al., 2020). The antibacterial properties and consequently activity of honey can differ between products based on factors such as bee species, nectar source, geographical location, environmental conditions, processing and storage techniques. However, the general consensus is that honey’s antibacterial activity is due to more than it’s physicochemical properties, and rather is facilitated by the combined effect of honey’s bioactive compounds, a factor suggested to represent honey’s true quality, and even proposed to be adopted as the international standardization rather than the current international honey quality standards (Majtan et al., 2021). Some resources categorize honey’s antibacterial activity as hydrogen peroxide (H_2_O_2_)-mediated versus non-peroxide mediated (Girma et al., 2019; Nader et al., 2021). Manuka Honey (MH) in particular, is well-characterized as a non-peroxide honey, with its broad-spectrum antibacterial properties, primarily attributed to its substantial methylglyoxal (MGO) component (Mavric et al., 2008; Carter et al., 2016; Cokcetin et al., 2016). MH’s MGO activity has been trademarked as the unique manuka factor (UMF^®^), graded to represent antibacterial potency the higher the UMF value is (Cokcetin et al., 2016). However, some studies have reported inconsistencies between the association of UMF values with antibacterial efficacy (Girma et al., 2019; Yu et al., 2020). While MGO is regarded as MH’s key antibacterial component, studies suggest that MGO is not MH’s only antibacterial element. In fact, innate bacterial processes are capable of detoxifying MGO thereby impeding its effects. Nonetheless, MH’s antibacterial effects are sustained, achieved by the sum of its unique constituents (Girma et al., 2019; Bouzo et al., 2020).

While antibiotic combination therapy has demonstrated significantly beneficial outcomes, resistance persists, among other challenges. The use of other known antimicrobial products in combinations with or without antibiotics offers an alternative strategy for exploration, with the hope of impeding the pace currently set by ABR. Some examples of studies evaluating the combinatorial effects of MH with antibiotics have reported the reversing of MRSA resistance to oxacillin, and the prevention of *S. aureus* resistance against rifampicin (Sherlock et al., 2010; Müller et al., 2013; Hayes et al., 2018; Liu et al., 2018). MH thus offers a promising alternative, both as a single multi-component agent in its own right, and in combination with antibiotics.

This study evaluated the efficacy of MH in combination with three antibiotics in mitigating growth of three staphylococci and their corresponding SCVs. While most studies using MH have focused on *S. aureus*, this study broadened the scope of the investigation to include two CNS, *S. epidermidis and S. lugdunensis*, owing to their rising clinical implications in biofilm-related infections, and consequently their contribution to the ABR crisis. Gentamicin (GEN), rifampicin (RIF), and vancomycin (VA) were employed in this study owing to their clinical relevance and increased reports of resistance (Bals and Filipescŭ, 1969; Dowding, 1977; Marais et al., 2009; Forrest and Tamura, 2010; Xiao et al., 2011; Rahimi, 2016; Wang et al., 2019). To the best of our knowledge, there has been no direct research into the effects of MH on SCVs at the time of this investigation. To test survivability and tolerance of this problematic phenotype, staphylococcal SCVs from all three species were subjected to varied concentrations of MH to further evaluate the efficacy of MH on their development and subsequent growth.

## 2 Materials and methods

### 2.1 Bacterial growth

*S. aureus* (ATCC^®^ 25923™), *S. epidermidis* (ATCC^®^12228™), and *S. lugdunensis* (ATCC^®^ 43809™) were used in this investigation. Stock samples of pure cultures were regularly maintained on tryptic soy agar (TSA) grown for 24 hrs at 37°C and stored at 4°C. Rapid purity and species identification tests were performed throughout this investigation using a range of standard methods: colony morphology, haemolysis pattern (on sheep blood agar (BA)), Gram stain, and API^®^ Staph (biomérieux). PCR (16s rRNA) was also performed to confirm the species identity of the generated SCV isolates (as non-contaminants despite atypical characteristics) (Sakai et al., 2004; Onyango et al., 2013).

### 2.2 Manuka honey & antibiotic preparations

Commercially available Manuka honey (MH) (UMF™ 20+, MGO 830+; Kiva health) was used in this study. This was stored in the dark at 4°C from which fresh stock solutions were made for each assay. Stock solution was aseptically prepared by dissolving 10 g of MH in 20 ml prewarmed Mueller-Hinton broth (MHB) to obtain a final stock solution of 50% (w/v). Stock MH was diluted further in MHB to obtain MH concentrations of 5 – 14% (w/v) (with 1% increments). Stock antibiotic preparations of gentamicin (GEN) and vancomycin (VA) (Avantor^®^, Canada) were prepared by dissolving 20 mg of antibiotic powder in 10 ml of MHB to obtain a final stock solution of 2 mg/ml. Rifampicin (RIF) (Avantor^®^, Canada) preparations were concurrently prepared in 10 ml of methanol (was a better solvent for this antibiotic than MHB) (Bodaghabadi et al., 2018; Subramaniam et al., 2019). Antibiotic stock solutions were diluted to final concentrations of 0.004 μg/ml – 15.63 μg/ml (two-fold dilution series) in MHB (sterile water for RIF) (Wiegand et al., 2008; Tan et al., 2009).

### 2.3 Determination of the minimum inhibitory concentration (MIC)

MIC determinations for WT *S. aureus, S. epidermidis*, and *S. lugdunensis* to MH and the three antibiotics were performed *in vitro* using the broth microdilution technique according to the CLSI and EUCAST guidelines (Wiegand et al., 2008). In a sterile flat-bottom 96-well microtiter plate (Corning^®^, USA), 100 µl of fresh log phase bacterial suspension adjusted to 10^3^-10^5^ cfu/ml was added to either 100 µl of premade MH or antibiotic at the aforementioned ranges. Absorbance was measured (580 nm; Multiskan Go, Thermo Scientific™) at 0hrs and 24hrs following incubation at 37°C (shaking was performed prior to OD (580 nm) readings). The MIC of MH and the MIC of antibiotics were recorded as the lowest concentrations without significant change in OD after 24 hrs. MICs were also confirmed visually (lowest concentrations with no visual turbidity or visible bacterial growth) after 24 hrs.

### 2.4 Effects of MH+ antibiotics on the growth of WT Staphylococci

The combinatorial effects of MH + antibiotics (0-0.975 μg/ml for antibiotics; 0-10%w/v for MH) against WT *S. aureus, S. epidermidis*, and *S. lugdunensis* were investigated using the checkerboard microdilution assay (Kamble et al., 2022) with minor revisions. In a 96-well microtiter plate, fresh premade solutions of 50 μl MH+ 50 μl antibiotics (at their varying dilution series) were added into each well. 100 μl of adjusted log phase bacterial suspension was added to each MH + antibiotic well and plates incubated at 37°C for 24 hrs (shaking was performed prior to readings). Growth patterns were recorded both spectrophotometrically (OD 580 nm) and visually as previously described. Fractional inhibitory concentration (FIC) and FIC index (FICI) were determined according to the following formula:


FICI=∑FIC of MH + FIC of antibiotic

$$\text{FIC of antibacterial product }\left(\text{MH or antibiotic}\right)=\frac{\text{MIC of antibacterial products in combination}}{\text{MIC of antibacterial product alone}}$$

The combinatorial effects were reported as synergistic (FICI ≤0.5); partially synergistic (FICI >0.5-1); additive (FICI 1-2); indifferent (FICI 2-4); or antagonistic (FICI >4) (Kamble et al., 2022).

### 2.5 SCV induction assays

Antibiotic chemotherapy is known to induce SCVs both *in vitro* and *in vivo*. In this investigation, GEN, RIF, and VA were individually assessed for their *in vitro* induction of SCV in the three staphylococcal isolates as previously described (Onyango et al., 2013). Briefly, fresh log phase bacteria (OD 0.05, 580 nm) were added to MHB-PBS solution (20%-80%, respectively). The different antibiotics were added to this medium to obtain increasing MICs (2X, 5X, 10X, 100X, and 1000X species MICs). Untreated controls of WT cultured in MHB-PBS were concurrently run. The cultures were incubated at 37°C for a duration of 10 days, with daily assessments for SCV development. SCV investigations were conducted by plating 100 µl of the treated cultures onto TSA and BA, and incubating for up to 72 hrs. The numbers of both WT and SCVs were counted and %SCV for the three staphylococci was calculated. Colonies were identified as SCVs if they grew > 24-72 hrs, were <1 mm in size, and displayed reduced pigmentation and haemolysis (on BA), in comparison to their corresponding WT (Onyango et al., 2013; Tuchscherr et al., 2016). SCVs were classified as either stable or transient, by sequential subculture (up to 3 passages) on TSA and BA for up to 72 hrs (Onyango et al., 2013). SCV transiency was characterized by observed reversion to WT phenotype upon subculture on stress-free media (TSB and BA). Colonies that maintained the SCV phenotype on stress-free media were characterized as stable and select isolates were tested in triplicate for species confirmation by PCR of the 16SrRNA.

### 2.6 SCV time-kill assay

Results from the SCV induction assay showed that while all three antibiotics were capable of inducing SCVs (transient or stable SCVs), GEN was found to be the only antibiotic to consistently induce stable SCVs. Only stable SCV phenotypes were selected for use in subsequent SCV-MH assays. A time-kill kinetic assay was performed to test the ability of MH-GEN combination to suppress the development of staphylococcal SCV via antibiotic induction (Tuchscherr et al., 2016; Kamble et al., 2022). In brief, MH at the respective species MIC was added to the MHB-PBS solution containing log-phase (OD 0.05, 580 nm) WT of all three species and antibiotics at the concentrations shown to induce stable SCVs, at time 0. Untreated cultures were used as a control. Cultures were monitored for both WT and SCV development as described in the previous section. The cfu/ml was plotted against time to obtain the time-kill curves (Tuchscherr et al., 2016; Kamble et al., 2022).

### 2.7 The effect of MH on SCV growth

In this investigation, stable SCVs were cultured with MH alone to assess its antibacterial activity on the growth propagation of SCVs. 20 stable SCV colonies were suspended into 2.5 ml of MHB, and 100 μl aliquots of this preparation added to 100 μl of MH (6-10% for *S. aureus*, 7-11% for *S. epidermidis*) in a 96-well plate. The absorbance was measured at 1hr intervals for up to 72 hrs. *S. lugdunensis* SCVs analyses were performed on Mueller-Hinton agar (MHA) plates due to challenges in detecting growth absorbance using microplate spectrometry. MHA plates adjusted to 6-10% (w/v) MH were prepared and inoculated with 100 μl aliquot suspensions of stable SCVs. Growth was visually monitored every 24 hrs for a duration of 72 hrs.

### 2.8 Statistical analysis

Unless otherwise indicated, all investigations were performed in triplicate on three separate occasions (n=9) with the corresponding controls. Data were analyzed and mean ± SD was calculated. One-way analysis of variance (ANOVA) was employed to assess the mean differences among and between the groups. P-value ≤ 0.05 was considered significant. Dunnett’s test was performed *post hoc* for all assays where p< 0.05.

## 3 Results

### 3.1 Antibiotic and Manuka honey MIC determination

Antibiotic MIC values for *S. aureus, S. epidermidis*, and *S. lugdunensis* ranged between 0.06-0.97 μg/ml. In general, of the three antibiotics tested, these staphylococci were most susceptible to RIF (0.06-0.24 μg/ml), followed by GEN (0.12-0.49 μg/ml), and lastly VA (0.49-0.96 μg/ml). The MICs of MH for the three staphylococci were 7%, 7-8%, and 6-7% (w/v), respectively (**Table 1**).

**Table 1:** Minimal inhibitory concentration (MICs) for WT *S. aureus, S. epidermidis*, and *S. lugdunensis*.

Isolate	WT MICs				MH + Antibiotic					
	MH (%, w/v)	GEN (μg/ml)	RIF (μg/ml)	VA (μg/ml)	MH + GEN		MH + RIF		MH + VA	
					FICI	Effects	FICI	Effects	FICI	Effects
***S. aureus***	7	0.49	0.24	0.96	1.50	A	0.92	PS	0.64	PS
***S. epidermidis***	7-8	0.12	0.06	0.96	1.13	A	1.14	A	1.50	A
***S. lugdunensis***	6-7	0.24	0.06	0.49	0.93	PS	1.50	A	1.50	A

MH, manuka honey; GEN, gentamicin; RIF, rifampicin; VA, vancomycin; FICI, Fractional inhibitory concentration index; Effects: A, additive; PS, partial synergy.

MIC was evaluated for both manuka honey (MH) and antibiotics separately, and in combination. FICI were also determined by the checkerboard microdilution assay. Results were interpreted as synergistic (FICI ≤0.5); partially synergistic (FICI >0.5-1); additive (FICI 1-2); indifferent (FICI 2-4); or antagonistic (FICI >4).

### 3.2 Susceptibility of wild-type staphylococci to combinatorial treatments of manuka honey and antibiotics

Antibacterial activity of MH in combination with each of the three antibiotics GEN, RIF, VA, yielded additive or partially synergistic results when tested against WT staphylococci of *S. aureus, S. epidermidis*, and *S. lugdunensis* in this study. In general, the combination of MH + antibiotic demonstrated better bacterial susceptibility than when the antibiotics were employed alone (**Table 1**).

For *S. aureus*, both MH+RIF and MH+VA combinations lowered the antibiotic MIC by at least 3-fold, which yielded partial synergy (FIC=0.92 and 0.64, respectively), while the MH+GEN combination lowered MIC 2-fold, which resulted in an additive effect (FIC=1.5). For *S. epidermidis*, the combination of MH +RIF, MH+VA, and MH+GEN all demonstrated additive effects, with FIC of 1.14, 1.5, and 1.13, respectively. The antibiotic MIC values for this species were lowered 4-fold (GEN), 3-fold (RIF) and 2-fold (VA) when MH was added. For *S. lugdunensis*, MH +GEN combination demonstrated partial synergy (FIC=0.93) with MIC lowered 3-fold, while the effects of MH in combination with RIF or VA were both lowered 2-fold, with FIC indicating an additive effect of 1.5 for both (**Table 1**).

### 3.3 Gentamicin-induced staphylococcal SCV

**Figure 1:**
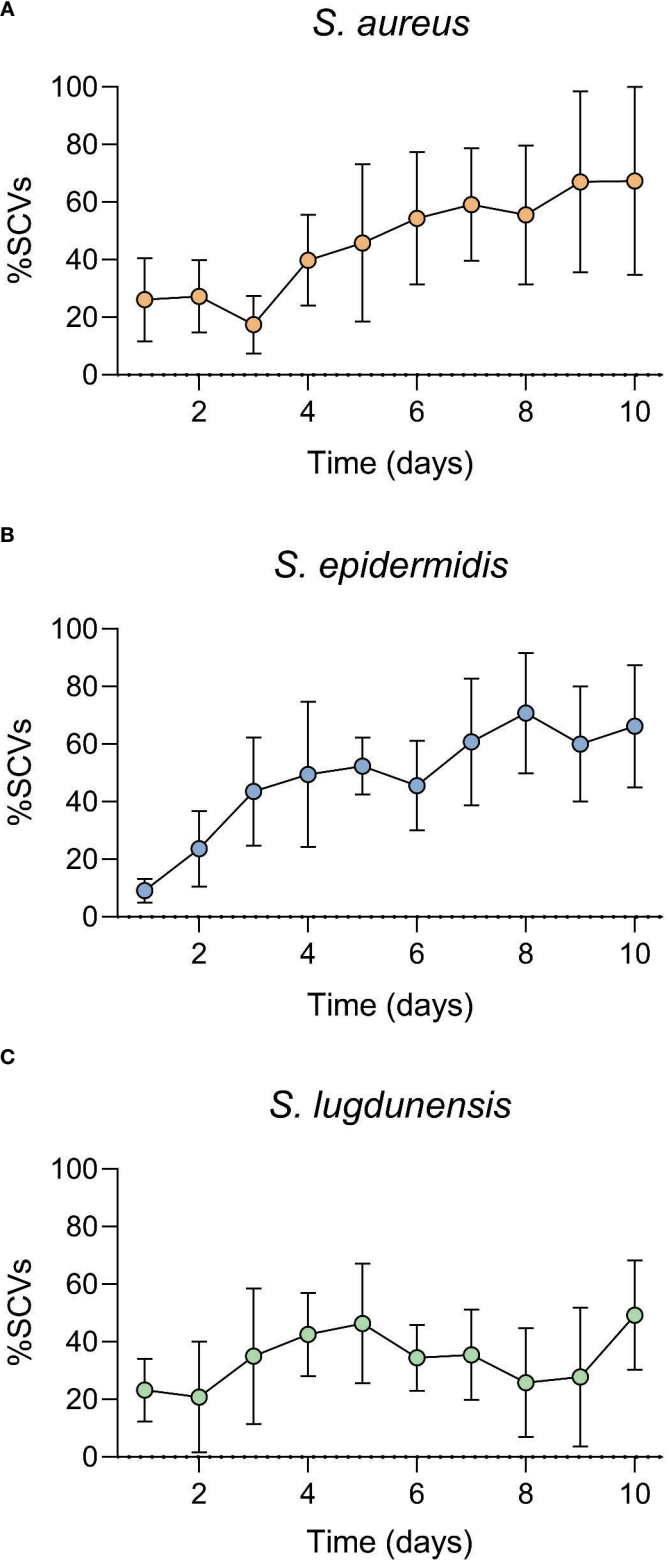
Staphylococcal small colony variant (SCV)-antibiotic induction assay over a 10-day incubation period. Gentamicin (GEN) was used as the induction agent at **(A)**. 10X MIC (*S. aureus*), **(B)**. 5X MIC *(S. epidermidis)* and **(C)**. 10X MIC (*S. lugdunensis).* SCVs numbers increased as exposure duration increased. SCV numbers represented here include both transient and stable SCVs observed during the 10-day incubation period.

**Figure 2:**
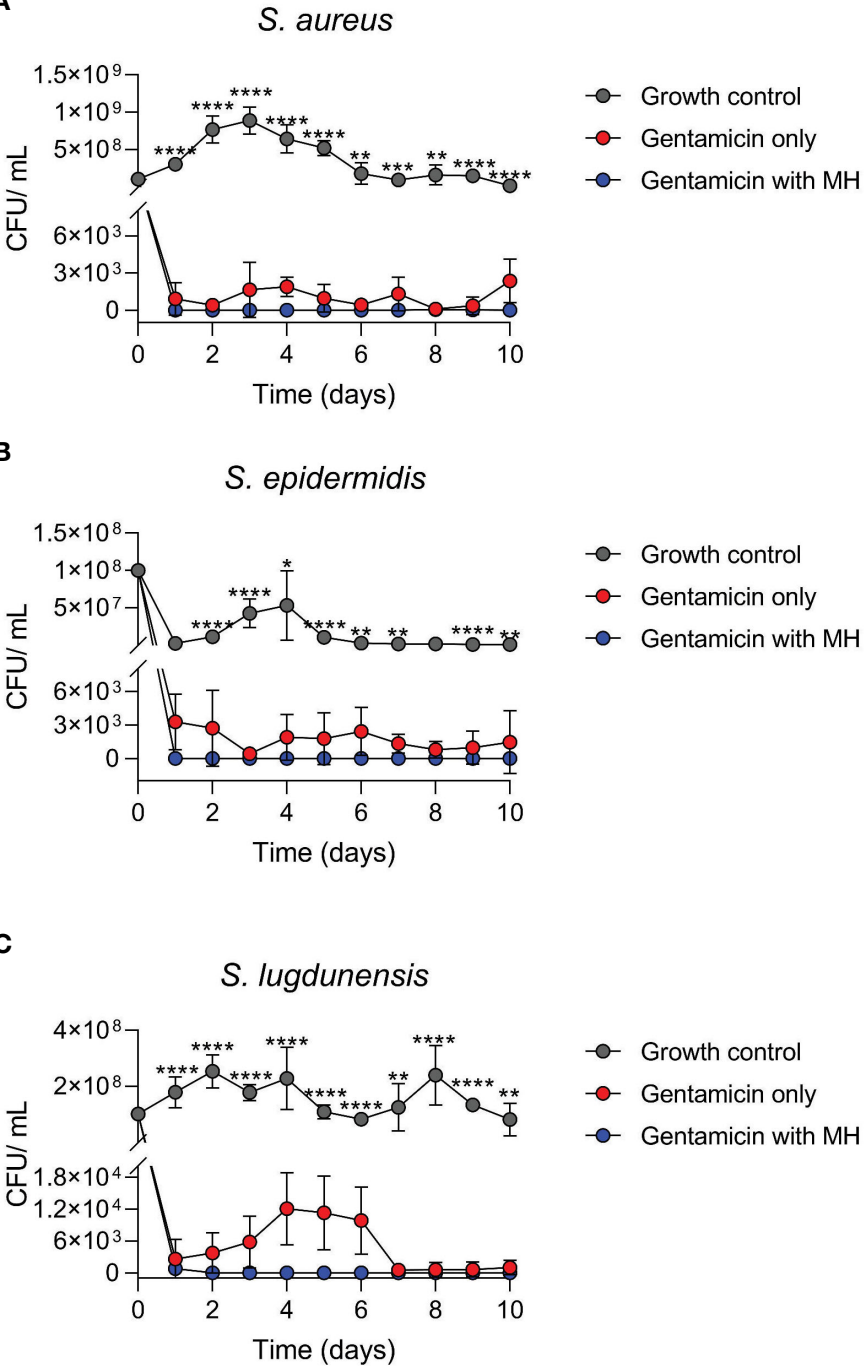
Time-kill assays of log-phase wildtype (WT) staphylococci in the presence of antibiotic alone (GEN) versus GEN in combination with manuka honey (MH). Cultures of *S. aureus* **(A)**, *S. epidermidis* **(B)**, and *S. lugdunensis* **(C)** were incubated for 10 days. MH at 7%, 8%, and 7% (w/v) was added to GEN at 10X, 5X, and 10X MIC for *S. aureus, S. epidermidis*, and *S. lugdunensis*, respectively. Colony-forming unit per ml (cfu/ml) represents both WT and SCVs observed daily. Figures are shown in cfu/ml versus time due to no growth on most days under MH + GEN treatment (log0 is undefined). One-way ANOVA statistical analysis was conducted over the 10-day timeline” and p values were defined for each time point. **** and *** indicate p <0.0001 and <0.001, whereas ** and * represent p values less than 0.01 and 0.05. Only significant results are depicted.

All three antibiotics (only at concentrations higher than MIC) were capable of inducing SCVs (transient or stable SCVs) in all three staphylococci during the 10-day *in vitro* induction period. A shift in population dynamics was observed as the incubation period progressed, with the number of SCVs in culture increasing as that of WT decreased during this time (**Figures 1**, **2**). GEN was the only antibiotic that consistently induced stable SCVs, which were observed at 10X (4.9 μg/ml), 5X (0.6 μg/ml), and 10X (2.4 μg/ml) GEN MIC for *S. aureus*, *S. epidermidis*, and *S. lugdunensis*, respectively. SCVs were characterized as <1mm colonies, with reduced pigmentation and haemolysis (on BA). The ability of MH to inhibit the *in vitro* development of antibiotic-induced SCVs was also investigated. The addition of MH yielded no observable SCVs (stable or transient) in all three staphylococci during the incubation period.

### 3.4 Time kill assays

This experiment compared the antibacterial activity of the antibiotic GEN alone, versus the combination of GEN+MH against WT log phase growth. In general, the results of the time-kill investigation showed that over the 10-day incubation period, treatment of all three staphylococci with the combination of GEN+MH resulted in a decline of at least 3 log in comparison with cultures exposed to GEN alone (**Figure 2**).

Cultures of *S. aureus* exposed to GEN+MH (10X MIC +7%) treatments showed a 3 log_10_ reduction in growth versus cultures of *S. aureus* exposed to GEN (8 cfu/ml vs 1045 cfu/ml, respectively, over 10 days) (**Figure 2A**). For *S. epidermidis* cultures exposed to GEN+MH (5X MIC +8%) showed 100% growth suppression in comparison to an average growth rate of 1715 cfu/ml in the presence of GEN alone (**Figure 2B**). For *S. lugdunensis* cultures, GEN+MH (10X MIC +7%) showed a 2 log_10_ decline in growth compared to GEN only cultures (4826 cfu/ml vs 78 cfu/ml) (**Figure 2C**).

**Figure 3:**
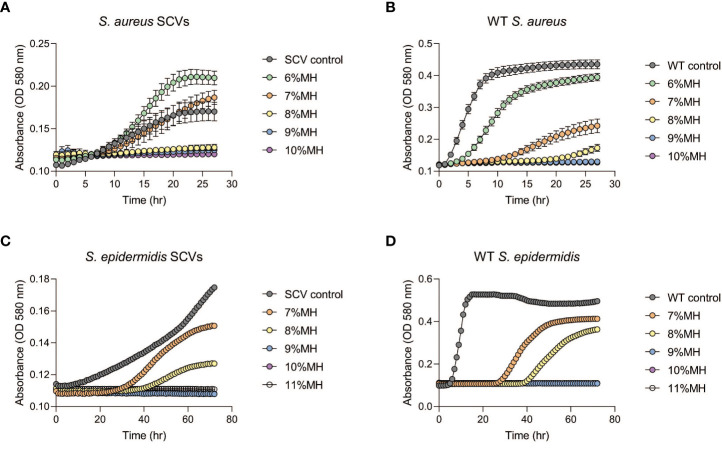
Staphylococcal SCVs and WT growth assays in the presence of manuka honey (MH). Stable SCVs recovered from SCV-induction assays and their WT counterparts were grown in Mueller -Hinton broth (MHB) with 6-10% (w/v) MH for *S. aureus* (**(A)**: SCVs; **(B)**: WT) and 7-11% (w/v) for *S. epidermidis* (**(C)**: SCVs; **(D)**: WT) for a duration of 24-72hrs. As MH concentrations increased, SCV growth progression was prolonged (indicated by low absorbance readings), and in some instances growth was completely inhibited (no change in absorbance over time). Growth of *S. lugdunensis* SCVs was undetectable using spectrophotometric analysis and was therefore conducted by growth on agar (see **Table 2**).

Following the observation that MH could inhibit the development of antibiotic-induced SCVs, this investigation tested whether MH alone could impede further growth of stable SCVs in culture. MH (6- 11% (w/v)) was added to the harvested stable SCVs from the SCV-induction assay and the growth patterns monitored spectrophotometrically in MHB (*S. aureus* and *S. epidermidis*) and on MHA (*S. lugdunensis*). **Figure 3** shows that in *S. aureus* and *S. epidermidis* instances, the addition of MH resulted in significantly longer lag phases for SCV cultures in comparison to their WT counterparts under the same conditions. In addition, the inhibition of SCVs of all three species was observed at MH concentrations higher than WT MIC (MH ≥8% vs 7% MH; ≥9% vs 7-8%; and MH ≥10% vs 6-7% for *S. aureus*, *S. epidermidis*, and *S. lugdunensis*, respectively) to achieve the same inhibitory effects (**Figures 3A–D**; **Table 2**). It was unexpected that SCV inhibition could be achieved by as little as 1% higher MH concentrations than their corresponding WT MH MIC, as was seen in *S. aureus* and *S. epidermidis*. We expected this to be much higher given the highly tolerant nature associated with SCVs. Interestingly, despite *S. lugdunensis* SCV growth being undetectable using spectrophotometric analyses at the same MH concentrations, plate cultures of the same were viable and showed that the stable SCV phenotype was maintained in the presence of MH at concentrations of up to 8%(w/v). The identity of these SCV isolates was confirmed as *S lugdunensis* using PCR.

**Table 2:** *S. lugdunensis* SCVs growth in the presence of MH.

Time (hrs)	Control	6%MH	7%MH	8%MH	9%MH	10%MH
**24**	**+**	**+**	**+**	**+**	**-**	**-**
**48**	+	**+**	**+**	**+**	**-**	**-**
**72**	+	**+**	**+**	**+**	**+**	

Stable SCVs isolated by antibiotic induction were incubated with MH at varying concentrations (6%-10%(w/v)) on Mueller-Hinton agar. Growth was visually monitored at 24, 48, and 72 hrs of incubation, with no phenotype switching observed (no WT). SCVs growth was indicated with the (+) symbol and the absence of SCV growth with the (-) symbol.

## 4 Discussion

In this current study, manuka honey (MH) was investigated as a potential alternative antimicrobial due to the challenges of antibiotic failure in the efficient resolution of staphylococcal infections, including the clearance of their SCVs in the presence of current antibiotic chemotherapeutics alone. RIF, GEN, and VA, routinely used in the clinical treatment of staphylococcal infections were tested in combination with MH on WT and SCVs of *S. aureus*, *S. epidermidis* and *S. lugdunensis.* Reports of increasing resistance patterns for these antibiotics demonstrates the need for alternative options (Bals and Filipescŭ, 1969; Dowding, 1977; Marais et al., 2009; Forrest and Tamura, 2010; Xiao et al., 2011; Wang et al., 2019). The use of combinations of antimicrobials with similar or different modes of action in clinical treatment of problematic infections is not new (García-Fuente et al., 2018; Mulani et al., 2019). Old antibiotics once rendered ineffective through bacterial resistance can now be ‘recycled’ or ‘repurposed’ through combinatorial therapy and have been used to achieve enhanced antibacterial activity and improved therapeutic gain against infections such as by MRSA and even biofilm populations.

This current study found that the combinatorial effects of MH + antibiotics yielded better antibacterial effects than when antibiotics alone were used. FICI results (>0.5-2) indicated an additive to partial synergistic activity for the three antibiotic + MH combinations tested (**Table 1**). Previous comparable studies testing the combinatorial effects of MH with clinically important antibiotics against staphylococci (*S. aureus* primarily), have mainly reported synergistic effects (FICI ≤0.5), with few others reporting partially synergistic and additive effects (FICIs ranging from 0.87-2) (Müller et al., 2013; Liu et al., 2015; Liu et al., 2018). The different results among the studies could be due to differences in bacterial isolates, antibiotic combination, and MH brands used. Nonetheless, collectively, this current study and others demonstrate that MH combinatorial treatment resulted in improved bacterial susceptibility than with antibiotics alone. MH as a whole exerts concerted antibacterial activity by the inhibition of different targets simultaneously. MH induces structural and morphological changes, alters the bacterial cell cycle and cell growth, modifies the physiological behavior of bacteria such as impeding iron acquisition through iron chelation, facilitates the irreversible alterations in bacterial membrane potential and membrane integrity, alters quorum sensing mechanisms, and disrupts other facets of bacterial metabolism (Henriques et al., 2009; Jenkins et al., 2011; Jenkins and Cooper, 2012; Maddocks et al., 2012; Jenkins et al., 2014; Ankley et al., 2020; Bouzo et al., 2020; Combarros-Fuertes et al., 2020). Interestingly, studies suggest that it’s the multifactorial antibacterial properties of this product that mitigates bacterial resistance development as the concerted actions likely overwhelm the bacterial stress responses, thereby impeding survival under the range of metabolic and cellular stressors (Carter et al., 2016).

In this study, it was postulated that the additive and partially synergistic results observed come not only from MH’s unique actions but could have also enhanced those exerted by the individual antibiotics. RIF is a potent bactericidal, exerting action through inhibition of bacterial mRNA transcription, but it also displays a heightened penetration capacity (Deresinski, 2009). In this study, MH’s action to irreversibly alter membrane potential and integrity may have been the first step in enhancing RIF’s uptake, thus hastening its penetrative capacity to its target, and together, their lethal actions facilitated a more efficient cidal effect. VA on the other hand is a glycopeptide that by binding to D-alanyl D-alanine, inhibits the synthesis and polymerization of peptidoglycan subunits necessary for viable cell-wall synthesis in Gram positive bacteria, like the staphylococci (Deresinski, 2009; Forrest and Tamura, 2010). The combination of VA+MH would exert a multifactorial effect on the metabolic activities of the external bacterial structures (wall weakening and exacerbated leakages), rapidly deteriorating the overall cellular integrity. GEN, a broad-spectrum aminoglycoside exerts cidal action by binding to the 16s rRNA at the 30s ribosomal subunit thereby inhibiting the synthesis of functional bacterial proteins (Beganovic et al., 2018). Other studies also suggest that the production of non-functional proteins may compromise the impermeability of the cell-wall, therefore the combined actions of GEN+MH would not only disrupt the functionality of this feature but effectively allow the antibiotic easy access to its internal target. GEN also induces the synthesis of hydroxyl radicals via the Fenton reaction due to protein depletion, and together with MH’s actions, increases hydroxyl radical production to induce cell death (Kohanski et al., 2007; Liu et al., 2015).

While this current study did not investigate staphylococcal resistance to MH, current literature suggests that it isn’t widespread (Combarros-Fuertes et al., 2020). Some studies show that MH’s concerted actions not only impair bacterial features and processes, but likewise impede specific bacterial antibiotic resistance mechanisms. For example, MH was suggested to affect the proton motive force in *Pseudomonas aeruginosa* which is necessary in generating energy to drive this bacterium’s proton-dependent drug efflux mechanism. Without a functional energy generator, antibiotic exportation (via efflux pumps) was reduced, which in addition to MH’s action to increase membrane permeability, greatly enhanced antibiotic activity, thereby rendering this crucial resistance mechanism ineffective (Bouzo et al., 2020) In summary, while MGO may be regarded as the prominent antibacterial constituent of MH, we postulate like other studies that the concerted effects of MH as a whole, deliver a more potent antibacterial action than its individual components alone. When used in combination with the selected antibiotics, the components of MH may elevate the antibacterial effect by not only compromising similar bacterial targets as the antibiotic, albeit via different mechanisms, but may aid the efficacy of an antibiotic’s unique action by providing easier access to internal targets such as ribosomal units through the initial cell entry processes. Nonetheless, more research exploring other combinatorial mechanisms is needed.

Staphylococci’s ability to form SCVs that display heightened persistence, extensive antibiotic tolerance, and hyper biofilm structures have complicated staphylococcal pathogenesis and exacerbates their role in ABR (Onyango et al., 2008; Onyango and Alreshidi, 2018). At the time this study was performed, no research to our knowledge had been conducted where MH was directly applied to SCVs. Comparable studies have involved biofilms of *S. aureus* and *P. aeruginosa* where MH was shown to prevent biofilm formation (Jervis-Bardy et al., 2011; Camplin and Maddocks, 2014; Lu et al., 2014; Kot et al., 2020). A 2014 study testing medical grade MH (Medihoney™, UMF or MGO content unknown) against *P. aeruginosa* biofilms reported the emergence of isolates with increased resistance to MH following treatment. The authors further described these isolates as slow growing and displaying increased antibiotic resistance and enhanced biofilm forming capacity. They postulated that these isolates could be SCVs (Camplin and Maddocks, 2014). As previously indicated, few other studies have reported bacterial resistance to MH, suggesting low prevalence, that has been attributed to its multifactorial action. Nonetheless, the observation by the 2014 study is interesting and warrants further investigation to ascertain if with increased usage, much like antibiotics, MH would present a selective pressure capable of inducing SCV phenotypes. Other studies have demonstrated the successful ability of MH to cause both cell death and the detachment of cells present within biofilms (Jervis-Bardy et al., 2011; Maddocks et al., 2012; Camplin and Maddocks, 2014; Lu et al., 2014; Piotrowski et al., 2017; Kot et al., 2020). SCV populations indeed share similarities with bacterial populations residing within biofilms including altered growth and metabolism, reduced expression of genes related to virulence factors, and heightened resistance to antibiotics. We therefore propose that MH could demonstrate similar antibacterial efficacy against SCVs cells. The observed efficacy of MH against biofilms from these past studies provide a basis for the potential effectivity of MH against SCVs directly in light of the close relationship between bacteria within biofilms and SCVs.

Similar to previous *in vitro* and *in vivo* studies (Kahl et al., 2016; Tuchscherr et al., 2016), this current investigation demonstrated GEN’s ability to induce SCVs at concentrations above WT MIC, a reflection of what is experienced when elevated and/prolonged antibiotic is administered in attempts to resolve recurring clinical infections. GEN is reported to select for and/or induce stable SCVs, that are characterized as electron transport deficient (von Eiff et al., 1998). The addition of MH to GEN induction experiments suppressed the growth of both WT and SCVs, with a reduction in total bacterial growth by >98% for all three species (**Figure 2**). This assay aimed to investigate the applicability of the simultaneous use of MH as an efficient countermeasure for the development of SCVs during infection control when elevated or prolonged GEN is administered. Our findings shows that no stable and/or transient SCVs were observed under the combinatorial use of GEN+MH suggesting that MH’s properties may interfere with the metabolic processes that induce SCV in the presence of this antibiotic. Further investigations exploring the exact mechanisms of this inhibitory effect would be needed. While transient SCVs were also isolated from the other antibiotic treatments, they were not tested against MH. We postulated that since MH demonstrated efficacy against both WT and stable SCV, MH would likely also inhibit the processes enabling temporary transition.

The extended lag phase of staphylococcal SCVs in comparison to their corresponding WT observed in this study (**Figure 3** extended approximately 10 hrs) is consistent with previous work which associated the long lag time of SCVs with their progression into heightened antibiotic tolerance (Vulin et al., 2018). Nonetheless, the SCV growth progression assays displayed successful suppression of growth for 24-72 hrs by varied concentrations of MH (**Figure 3**). The ability of MH to inhibit both WT and SCV phenotypes is highly significant since infection control is often complicated by the presence of SCVs and their ability to display phenotypic shift mechanisms (PSM). This mechanism enables WT to rapidly transition to SCVs under the pressures of antibiotics or other host’s immune factors, and subsequent reversion of SCV to WT phenotypes (such as in the case of transient SCVs) when pressures abate, resulting in the chronicity of infections (Onyango et al., 2013). To eliminate both phenotypes simultaneously, MH concentrations higher than WT MIC concentrations would be needed. To determine a suitable concentration that would achieve this outcome, we tested stable SCVs against MH (6-11%) using broth microdilution and observed an unexpected result. In *S. aureus* and *S. epidermidis* SCV-MH treatments (**Figure 3**), SCV inhibition could be achieved by as little as 1% higher MH concentrations than their corresponding WT MH MIC. We expected this to be much higher given the persistent and exceedingly tolerant reputation of SCVs. However, with *S. lugdunensis* SCVs cultures, these SCVs remained stable and could persist in some instances up to 9% MH (2-3% higher than *S. lugdunensis* WT MIC). We therefore concluded that for complete clearance of both WT and SCVs to be achieved simultaneously using MH, concentrations >3% that of their corresponding WT MH MIC would produce the best results. We postulate that SCV’s altered metabolism, exhibited by slower growth, a characteristic known to impede antibiotics that depend on WT metabolism, may also hamper other antimicrobials. Therefore, to achieve adequate SCV clearance, both higher and prolonged MH exposure would be the most effective. While it remains unclear why there would be variances in *S. lugdunensis* detection, this experiment reiterated the importance of using both broth and agar techniques concurrently to confirm antibacterial effects especially when working with SCVs.

While medical grade MH is primarily used as a topical ointment (gels), or incorporated into wound dressings, other clinical applications have been documented. A recent study investigated the therapeutic potential of intravenously administered MH to treat septicaemia (caused by MRSA) and New Delhi Metallo-β-lactamase (NDM)-1 producing *Klebsiella pneumoniae* in a mouse model. All MRSA-infected mice recuperated after IV treatment with MH, and a substantial reduction of bacterial load was seen in K. *pneumoniae* subjects (Qamar et al., 2018). Other more obscure yet interesting uses include treatment of dry eye (Hu et al., 2022), dental ailments (Al-Kubaisi and Al-Ghurabi, 2023; Opšivač et al., 2023), and sinus rinses for cystic fibrosis patients (Roberts et al., 2022). To improve MH’s delivery to problematic sites of infection, circumvent its dilution, and improve contact time, other studies have explored the use of agents such as artificial liposomes and micelles for the *in vivo* delivery of MH (Maddocks and Jenkins, 2013; Patra et al., 2018). MH microneedles that are minimally invasive, penetrating about 50–100 µm into the skin without affecting the nerves, and simultaneously achieve efficient delivery of MH to sites of infection have also been explored (Frydman et al., 2020). Sustained *in vivo* contact would be especially advantageous in chronic scenarios against SCVs which demonstrate slow growth and would require extended contact time with MH for inhibition.

In conclusion, as the current clinical antibiotic arsenal is met with extensive bacterial resistance, and the lack of new and efficacious antibiotic options in the development pipeline, there remains an urgency for concerted efforts to develop novel and alternative measures to circumvent ABR and offer long-lasting clinical interventions. This study alongside others demonstrated the combinatorial effect of MH with three antibiotics, RIF, GEN, and VA in staphylococcal growth control. The addition of MH to antibiotics exerts a two-fold advantage – significantly improved bacterial growth control, and reduction of antibiotic concentrations administered. In addition, with minimal bacterial resistance to MH observed in other investigations, this non-antibiotic alternative stands as a viable alternative particularly in combinatorial therapy of staphylococcal infection, all of which improve the ABR dilemma currently plaguing humanity. These combined findings demonstrate MH’s ability to improve the sensitivity of all three staphylococci to antibiotics by lowering the antibiotic MIC values, which could help augment antibiotic potency. This subsequently would prolong the shelf life of these antibiotics, and in turn alleviate the progression of ABR where staphylococci are involved. With technological advancements steadily growing, there’s potential to expand the therapeutic applications of MH as an alternative treatment in problematic infection control. While *in vitro* testing demonstrates MH’s efficacy at low concentrations, more *in vivo* investigations are still needed to determine comparability. Additional clinical data is also required to support the efficacy and usage of MH in infection control.

## Data availability statement

The original contributions presented in the study are included in the article/**Supplementary Material**. Further inquiries can be directed to the corresponding author.

## Author contributions

All authors listed have made a substantial, direct, and intellectual contribution to the work, and approved it for publication.

## Supplementary material

Supplementary Figure 1 Visual comparison of size and pigmentation between WT and stable SCVs (harvested from GEN treatments). WT and SCVs of *S. aureus* **(A)** *, S. epidermidis* **(B)**, and *S. lugdunensis* **(C)** were cultured on tryptic soy agar (TSA) and incubated at 37°C for 24-72hrs (first row: whole plate; second row: zoom-in section. Pictures taken using the same scale). 24h and 72h pictures are shown for WT while only 72 h images are shown for SCVs as their colonies did not appear until 48-72 hrs of incubation.

Supplementary Figure 2 Examples of recovered plates from time-kill assay where MH at respective MIC was added to the induction media containing GEN. An aliquot of culture medium of *S. aureus* **(A)** *, S. epidermidis* **(B)**, and *S. lugdunensis* **(C)** were plated on TSA and incubated for 24-72 hrs at 37°C to observe possible induction of SCVs (first column: growth control; second column: recovered plate from GEN only treatments; third column: zoom-in sections; fourth column: recovered plate from GEN+MH treatments).

Supplementary Figure 3 Example images of *S. lugdunensis* SCVs growth in the presence of MH. Stable SCVs isolated from previous antibiotic induction assays were maintained on TSA. These were then sub-cultured in the presence of MH at varying concentrations (6%-10%(w/v)) on Mueller-Hinton agar to investigate the effect of MH on stable SCVs (**(A)**: whole plates; **(B)**: zoom-in sections). *S. lugdunensis* SCVs were capable of consistent growth at MH concentrations of <8%(w/v), indicating that their MIC would be >9%MH.
