# Supplementary figures and images for: Combinatorial efficacy of Manuka honey and antibiotics in the in vitro control of staphylococci and their small colony variants
Source: Front Cell Infect Microbiol. 2023 Oct 19;13:1219984. doi: 10.3389/fcimb.2023.1219984 (PMC10622673; doi:10.3389/fcimb.2023.1219984)

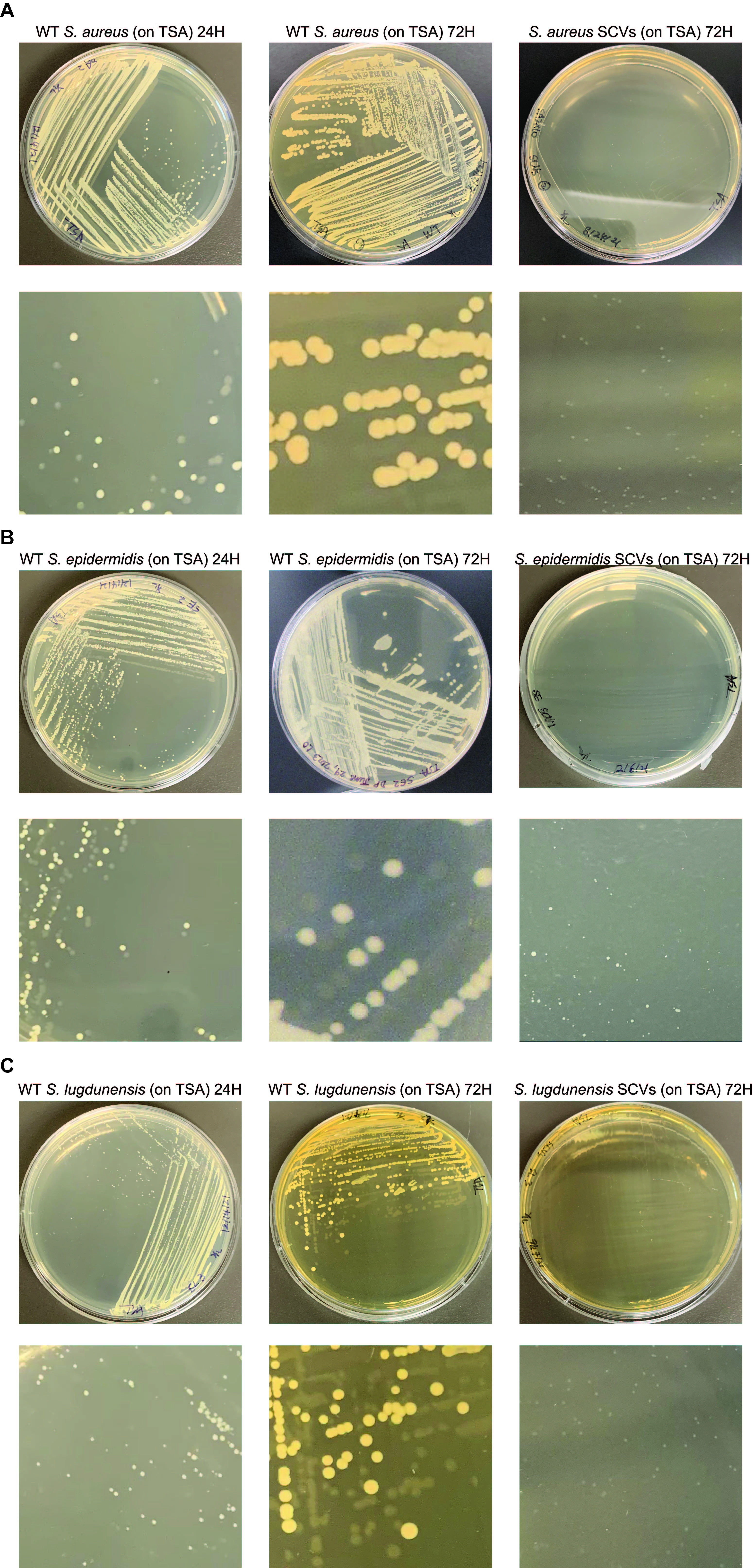

Supplement: Supplementary Figure 1 — Visual comparison of size and pigmentation between WT and stable SCVs (harvested from GEN treatments). WT and SCVs of S. aureus (A) , S. epidermidis (B), and S. lugdunensis (C) were cultured on tryptic soy agar (TSA) and incubated at 37°C for 24-72hrs (first row: whole plate; second row: zoom-in section. Pictures taken using the same scale). 24h and 72h pictures are shown for WT while only 72 h images are shown for SCVs as their colonies did not appear until 48-72 hrs of incubation. [file Image_1.jpg]

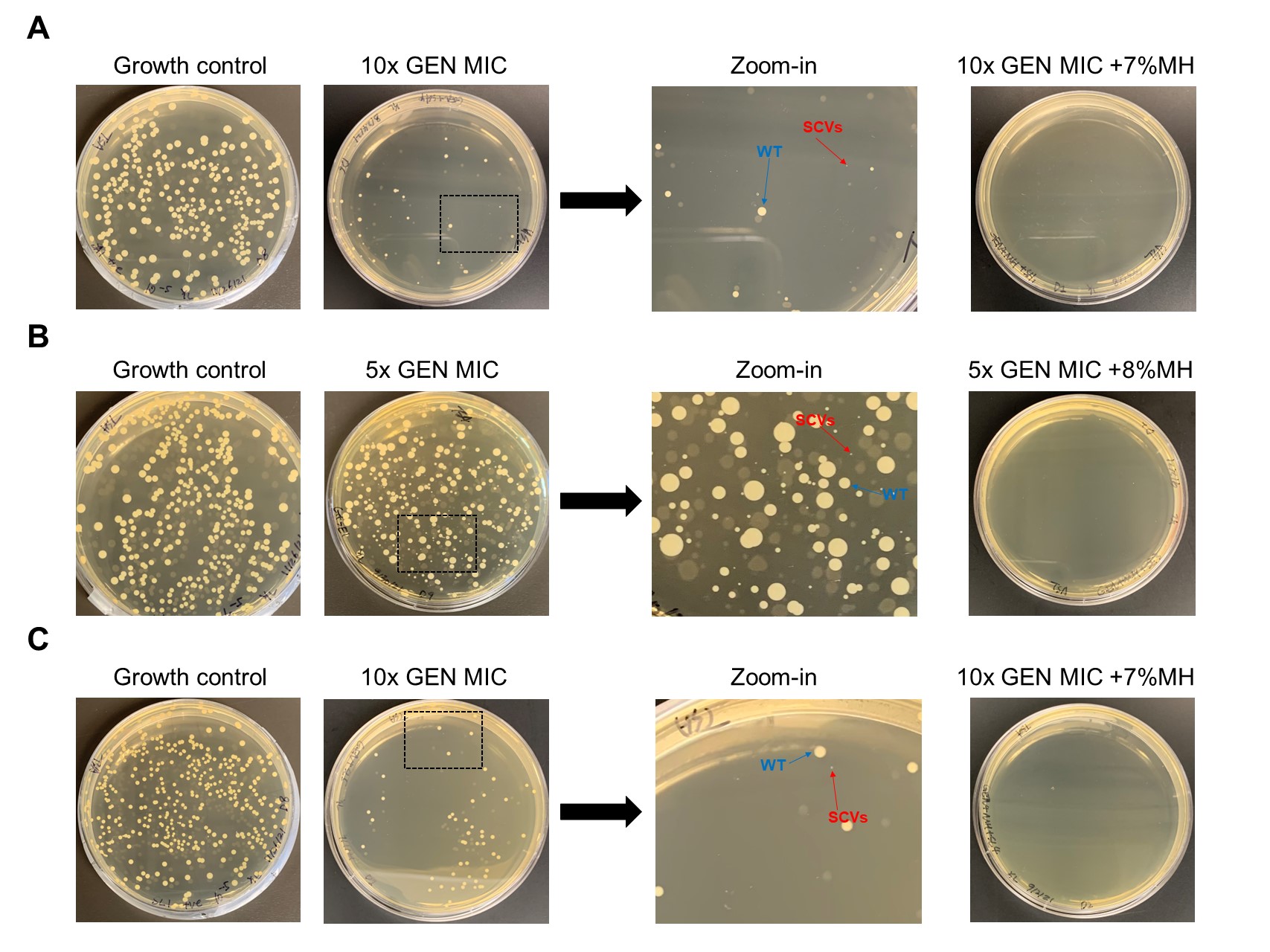

Supplement: Supplementary Figure 2 — Examples of recovered plates from time-kill assay where MH at respective MIC was added to the induction media containing GEN. An aliquot of culture medium of S. aureus (A) , S. epidermidis (B), and S. lugdunensis (C) were plated on TSA and incubated for 24-72 hrs at 37°C to observe possible induction of SCVs (first column: growth control; second column: recovered plate from GEN only treatments; third column: zoom-in sections; fourth column: recovered plate from GEN+MH treatments). [file Image_2.jpg]

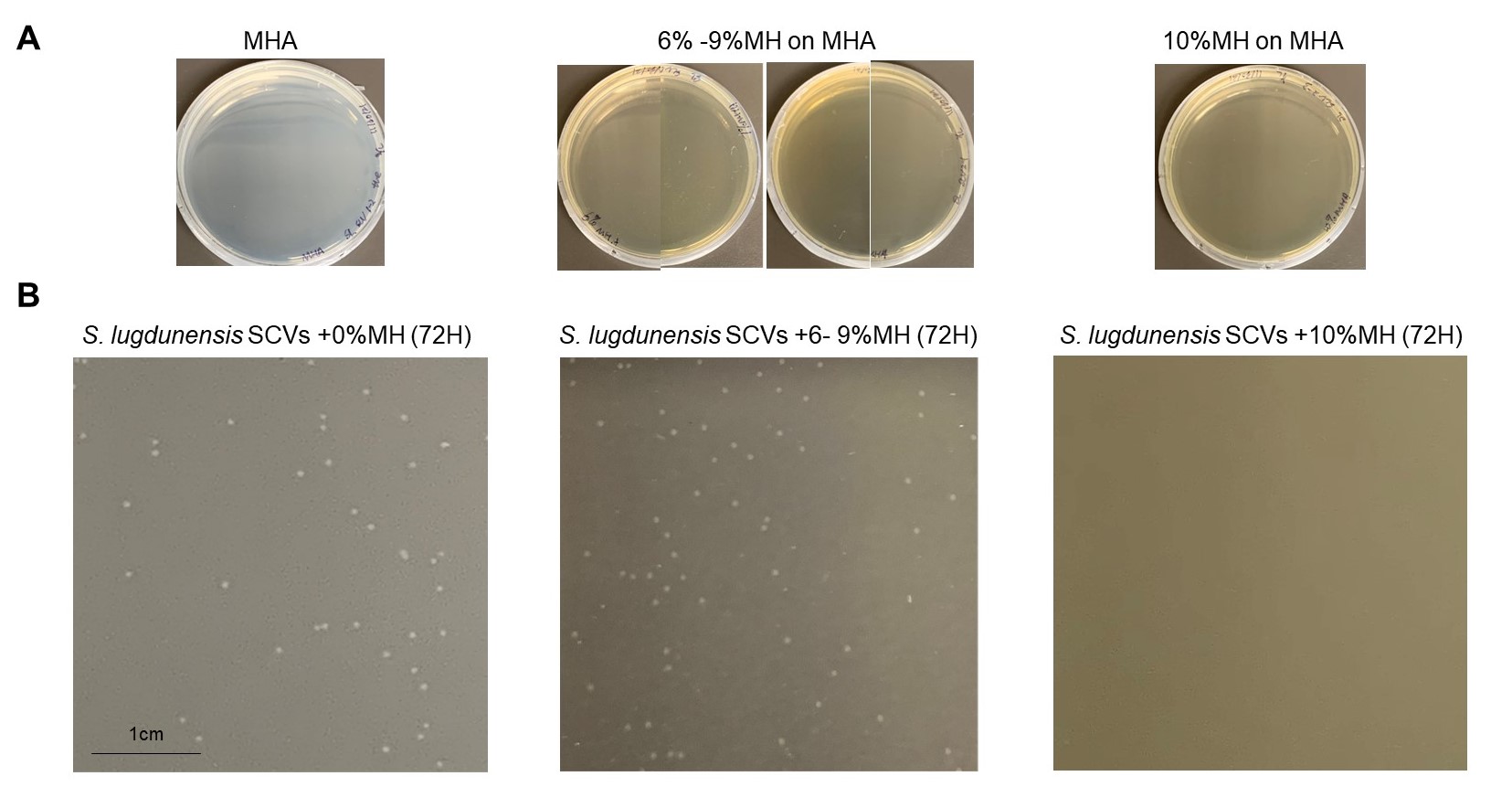

Supplement: Supplementary Figure 3 — Example images of S. lugdunensis SCVs growth in the presence of MH. Stable SCVs isolated from previous antibiotic induction assays were maintained on TSA. These were then sub-cultured in the presence of MH at varying concentrations (6%-10%(w/v)) on Mueller-Hinton agar to investigate the effect of MH on stable SCVs ((A): whole plates; (B): zoom-in sections). S. lugdunensis SCVs were capable of consistent growth at MH concentrations of <8%(w/v), indicating that their MIC would be >9%MH. [file Image_3.jpg]
